# Improved reproducibility of unit-cell parameters in macromolecular cryocrystallography by limiting dehydration during crystal mounting

**DOI:** 10.1107/S1399004714012310

**Published:** 2014-07-25

**Authors:** Christopher Farley, Geoffry Burks, Thomas Siegert, Douglas H. Juers

**Affiliations:** a Department of Physics, Whitman College, 345 Boyer Avenue, Walla Walla, WA 99362, USA; b Program in Biochemistry, Biophysics and Molecular Biology, Whitman College, 345 Boyer Avenue, Walla Walla, WA 99362, USA

**Keywords:** unit-cell reproducibility, cryocooling, humidity, dehydration

## Abstract

In macromolecular cryocrystallography unit-cell parameters can have low reproducibility, limiting the effectiveness of combining data sets from multiple crystals and inhibiting the development of defined repeatable cooling protocols. Here, potential sources of unit-cell variation are investigated and crystal dehydration during loop-mounting is found to be an important factor. The amount of water lost by the unit cell depends on the crystal size, the loop size, the ambient relative humidity and the transfer distance to the cooling medium. To limit water loss during crystal mounting, a threefold strategy has been implemented. Firstly, crystal manipulations are performed in a humid environment similar to the humidity of the crystal-growth or soaking solution. Secondly, the looped crystal is transferred to a vial containing a small amount of the crystal soaking solution. Upon loop transfer, the vial is sealed, which allows transport of the crystal at its equilibrated humidity. Thirdly, the crystal loop is directly mounted from the vial into the cold gas stream. This strategy minimizes the exposure of the crystal to relatively low humidity ambient air, improves the reproducibility of low-temperature unit-cell parameters and offers some new approaches to crystal handling and cryoprotection.

## Abstract 2

Unit-cell reproducibility is improved by maintaining the humidity of crystals during cryomounting.

## 1. Introduction   

Data collection at cryogenic temperature is the current method of choice for macromolecular structure determination *via* X-ray diffraction, especially at high-intensity synchrotron-radiation sources. Because radiation damage occurs more slowly at the low temperature, complete data sets can usually be obtained from single cryogenically cooled crystals. However, for weak signals and/or small crystals sufficient data often cannot be collected from a single crystal prior to the onset of radiation damage, necessitating the use of methods requiring multiple crystals. Although it had generally been assumed that increased non-isomorphism (relative to room temperature) from variation in the details of the cryocooling process would obscure weak anomalous scattering signals, recent work has shown that even sulfur SAD can be carried out using multiple cryogenically cooled crystals (Liu *et al.*, 2011 ▶, 2012 ▶). Hence, a key aspect of multiple crystal methods at cryogenic temperatures is the identification of groups of isomorphous crystals from which data may be successfully merged (Giordano *et al.*, 2012 ▶; Foadi *et al.*, 2013 ▶)

From the sample-handling side, more information about the aspects of crystal mounting that produce unit-cell variation could allow more precisely controlled cooling protocols, which would increase the probability that any two cryocooled crystals may be isomorphous. This would be helpful not only for the recently described multiple-crystal methods but also for more traditional approaches including multiple isomorphous replacement (MIR) and the use of *F*_o_ − *F*_o_ electron-density maps to identify bound ligands and conformational changes. Improved reproducibility in crystal characteristics after cooling (*i.e.* unit-cell parameters, diffraction limit and mosaicity) would also be helpful for the systematic identification of favourable cryocooling conditions for the crystal of choice.

Several different methods of mounting crystals for cryocrystallography have been discussed (Hope, 1988 ▶; Teng, 1990 ▶; Riboldi-Tunnicliffe & Hilgenfeld, 1999 ▶; Kitago *et al.*, 2005 ▶; Warkentin *et al.*, 2008 ▶; Warkentin & Thorne, 2009 ▶). The most common is to simply retrieve the crystal from its growth drop with a small nylon or polyimide loop, briefly expose it to some cryoprotective treatment and then cool it to cryogenic temperature (Teng, 1990 ▶). During the cryomounting process multiple decisions must be made that may or may not impact the cryocooling result, including choices of crystal size, loop size, cryosolution and soaking time in cryosolution; whether to eliminate the cryosolution surrounding the crystal; whether to cool by dunking the crystal into liquid or by placing it in a cold gas stream; for dunking, how fast to move the crystal into the liquid and whether to use hyperquenching (Warkentin *et al.*, 2006 ▶); and for gas-stream cooling, whether to block the cold stream prior to mounting on the goniometer. Other variables include the distance between the microscope and the cryosystem, the ambient humidity of the room, the humidity of the crystal buffer, the age of the crystals, the temperature and velocity of the cold stream, the temperature of the sample prior to cooling, the orientation of the crystal in the loop and the orientation of the loop relative to the velocity of the gas stream.

To date, there have been few studies of the above variables, especially with respect to the reproducibility of unit-cell parameters. Developments in cryomounting techniques have been targeted towards eliminating the need for post-crystallization cryoprotection treatments by reducing the amount of solution external to the crystal, by increasing the cooling rate and by cooling under high pressure. The volume of external solution can be limited with microfabricated mounts that facilitate wicking of solution from crystals (Thorne *et al.*, 2003 ▶), loopless mounting (Kitago *et al.*, 2005 ▶, 2010 ▶), aggressive removal of external aqueous solution with the crystal under oil (Warkentin & Thorne, 2009 ▶) and the complete wicking of solution from the crystal prior to cooling (Pellegrini *et al.*, 2011 ▶). The cooling rate can be increased by plunging into liquid propane (Walker *et al.*, 1998 ▶) or hyperquenching (Warkentin *et al.*, 2006 ▶). Cooling under high pressure prevents the formation of ice I and damage caused by the resulting volume expansion (Thomanek *et al.*, 1973 ▶; Kim *et al.*, 2005 ▶; Burkhardt *et al.*, 2012 ▶).

Here, we investigate some aspects of the cryomounting process to identify potential sources of unit-cell variation that may be reduced with more precise cooling protocols. Our results suggest that for thaumatin crystals a major source of unit-cell variation is evaporation of water during the mounting procedure, which is impacted by several variables, motivating some simple practices to improve reproducibility. We describe a new cryomounting strategy designed to limit water loss during transfer to the cryogen. The vial-mounting method reduces dehydration-based unit-cell variation and offers the possibility of systematic vapor-diffusion-based schemes for equilibration with cryoprotective agents that reduce crystal handling.

## 2. Methods   

### 2.1. Chemicals   

Unless otherwise noted, all chemicals were from Sigma–Aldrich (St Louis, Missouri, USA).

### 2.2. Crystals   

Crystals were grown at 296 K *via* hanging-drop vapor diffusion using 24-well plates (Hampton Research, Aliso Viejo, California, USA). Crystals appeared in a few days and were used within several weeks (thaumatin and lysozyme) or months (thermolysin) of growth. For tetragonal thaumatin (Sigma–Aldrich catalog No. T7638), the well solution was 0.3–0.9 *M* potassium sodium tartrate and the protein was at 35 mg ml^−1^ in 0.1 *M* HEPES pH 7.3 (Ko *et al.*, 1994 ▶). Unless growing on the surface of the drop or cover slips, the thaumatin crystals grew in a square bipyramid habit with a height:base ratio of ∼2.5:1. The crystal dimensions were measured either with the mounting microscope or with the diffractometer alignment microscope and the ‘crystal size’ reported here is the length of a side of the square-pyramid base. For tetragonal lysozyme (Sigma–Aldrich catalog No. L6876), the well solution was 20 m*M* sodium acetate pH 4.5, 5%(*w*/*v*) NaCl and the protein was at 80 mg ml^−1^ in 40 m*M* sodium acetate pH 4.5 (Forsythe *et al.*, 1999 ▶). For hexagonal thermolysin (Sigma–Aldrich catalog No. P1512), the well solution was 30% saturated ammonium sulfate and the protein was at 60–80 mg ml^−1^ in 50 m*M* MES pH 6.0, 0.5 *M* NaCl, 45%(*v*/*v*) DMSO (Hausrath & Matthews, 2002 ▶). Thaumatin and lysozyme were set up by mixing protein with well solution (total drop volume 8–9 µl), while for thermolysin the protein solution was used without mixing with the well solution (drop volume 6 µl). The cryoprotective solutions used for thaumatin were 35%(*w*/*v*) ethylene glycol (EG), 0.8 *M* potassium sodium tartrate and 3.5 *M* trimethylamine *N*-oxide (TMAO) with and without 0.1 *M* potassium sodium tartrate; that for thermolysin was 50%(*w*/*w*) sucrose and that for lysozyme was 35%(*w*/*v*) EG, 5%(*w*/*v*) NaCl, 20 m*M* sodium acetate pH 4.5.

### 2.3. Standard cryocooling   

Crystals were mounted using nylon cryoloops of 20 µm diameter with microtubes snapped at the 18 mm notch mounted on SPINE crystal caps (Hampton Research). To harvest the crystals, the cover slips were inverted, 10–20 µl of well solution was placed on the crystallization drop and any crystals growing on the surface of the drop were poked into the drop with the loop so that they were completely submerged. Crystals were then transferred with a loop into a 10–15 µl drop of cryosolution and soaked either in ambient air (30 s soak time) or with the drop placed over a well containing cryosolution (2 min or longer soak times).

Crystals were cooled and mounted on the diffractometer either by direct placement into the cryostream (Cryojet, Oxford Instruments, Abingdon, England; 100 K; sample flow 6 l min^−1^, shield flow 4 l min^−1^) or by plunging them into a foam dewar (Hampton Research) of liquid nitrogen and transferring them to the cryostream with CryoTongs (Hampton Research). In the typical condition, the loop width was chosen to be somewhat smaller than the crystal to achieve an intermediate amount of solution external to the crystal. Crystals were mounted by looping directly and then cooled without blotting or wicking any of the external solution remaining on the crystal. Thaumatin crystals were mounted with the long axis of the crystal parallel (the usual orientation) or perpendicular to the long axis of the loop. The lysozyme crystals were blocks and no attempt was made to obtain a systematic looping orientation. Thermolysin crystals were rods mounted with the long axis of the crystal parallel to the loop axis. For cooling in the gas stream, the loop was manipulated by hand. After looping, the crystal was placed on the gonio­meter without blocking the cold stream such that the velocity vector of the crystal was roughly perpendicular to the velocity vector of the cold stream. Two different microscope to cryostream distances were used (1.2 and 3.0 m). For cooling *via* plunging, the loop was manipulated with a CryoWand (Hampton Research), the dewar of liquid nitrogen was placed immediately adjacent to the microscope and the cold gas layer above the nitrogen was typically ∼1 cm. In some cases, the crystal was coated with oil prior to plunging it into liquid nitrogen (Immersion Oil Type B, Cargille Laboratories, Cedar Grove, New Jersey, USA). The crystal was then transferred to the gas stream using CryoTongs (Hampton Research). Ambient humidity and temperature were monitored with EL-WiFi-TH sensors (Lascar Electronics, Salisbury, England).

### 2.4. Vial mounting with humid flow   

#### 2.4.1. Humid flow   

**Figure 1:**
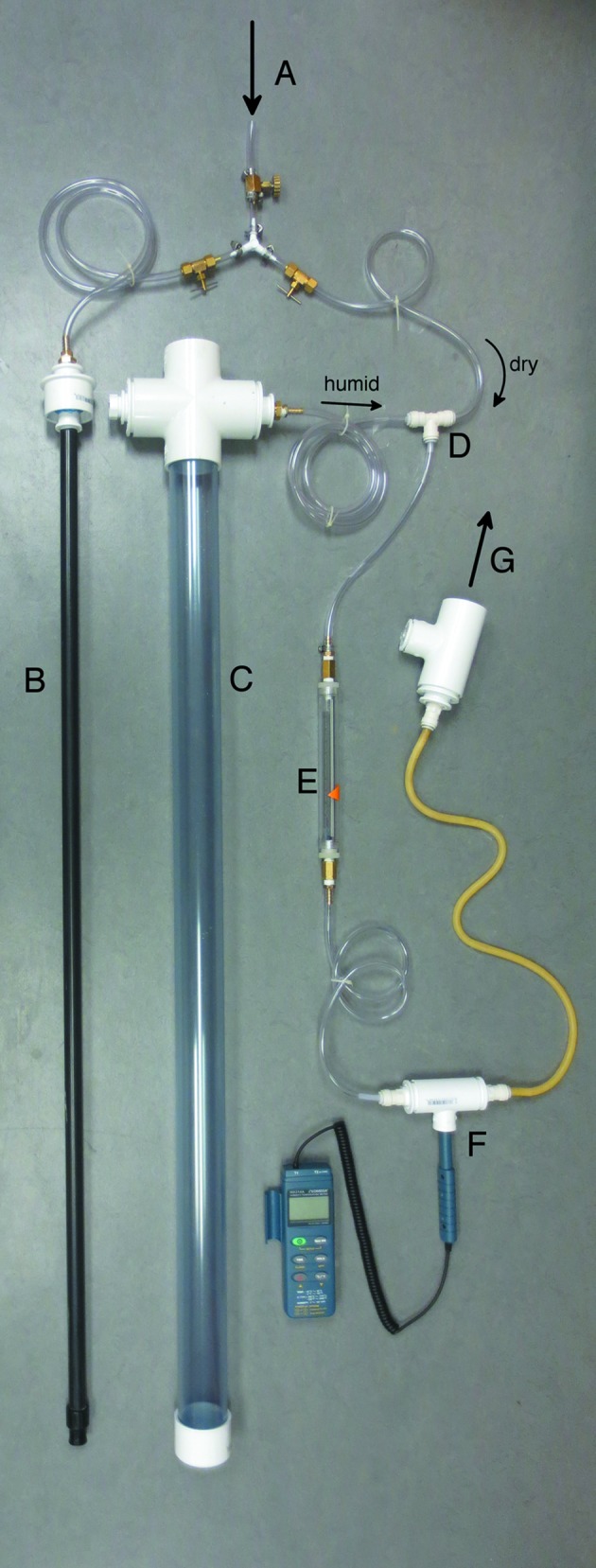
Humidity-control system. Pressure-regulated house air is divided into dry and humid paths (A). The latter flows through the black tube (B), exiting at the bottom of a water reservoir (C). (The black tube has been removed from the humidification chamber for clarity.) The air flows up through the water, becoming humidified, and then recombines with the dry flow (D). The flow rate (E) and humidity (F) are measured inline and the humid air exits a 1.9′′ diameter tube (G). See §[Sec sec2]2 for details.

Previously described devices for maintaining the humidity of macromolecular crystalline samples have been targeted at room-temperature data collection and focus on finely controlled and stable humidity over a small area (Kiefersauer *et al.*, 2000 ▶; Sjögren *et al.*, 2002 ▶; Sanchez-Weatherby *et al.*, 2009 ▶; Baba *et al.*, 2013 ▶). Here, we wish to humidify a larger area covering one or two cover slips and a vial, so the flow rate must be higher. Additionally, since we are using the humid flow for crystal mounting rather than data collection, long-term stability is less important. The humidity-control apparatus, which divides house air [relative humidity (RH) of ∼10%] into two paths, humidifies one and then recombines the two paths to yield a controllable humid flow, is built from off-the-shelf components (Fig. 1 ▶). The water reservoir, humidity probe chamber and output port were all fashioned primarily from schedule 40 PVC pipe and fittings (Dura Plastic Products, Beaumont, California, USA; Spears Manufacturing, Auburn, Washington, USA; the clear pipe was from US Plastic Corp., Lima, Ohio, USA). Water reservoir, 4–8′ long pipe (nominal diameter 2 or 3′′) with cap (bottom) and tee or cross (top); humidity probe chamber, reducing tee (1 × 1 × ½′′); output port, reducing tee (1½ × 1½ × 1′′). Topping the reservoir with a cross provides an extra port that can be used to replenish the water. Reducer bushings couple the chambers and output port to tubing [either one stage *via* slip/thread (½′′) or two stage, which is more modular, *via* outer slip/slip(1′′)–slip(1′′)/thread(½′′)]. For coupling to the air path in the reservoir, a specialized connector was made using two Dura Plastics pieces glued together: slip(2′′)/slip(1′′) and slip(¾′′)/thread(½′′), with a slip(1′′)/thread(½′′) friction-fitted at the very top to accept a tubing connector. The system is plumbed mainly with polyvinyl tubing (inner diameter ¼′′, outer diameter 3/8′′, pressure limit 55 psi), brass needle valves with compression fittings, brass hose barbs and quick connect fittings (Watts, North Andover, Massachusetts, USA). For flexibility, silicone tubing was used between the humidity probe chamber and the output port. The flow rate was monitored with an inline floating-ball flowmeter (Gilmont Instruments, Barring­ton, Illinois, USA) and the humidity was monitored with a humidity/temperature probe (HH314; Omega Engineering, Stamford, Connecticut, USA) coupled to the humidity probe chamber with a section of a 15 ml centrifuge tube. The humidification efficacy and sustainability depend on the details of the water reservoir and the delivery of air at the bottom of the reservoir. The work presented here used a 4′ long 2′′ diameter pipe for the reservoir and a 4′ long ½′′ diameter pipe terminated in a sprinkler head for the air path, yielding a maximum humidity of ∼95% for intermittent usage. With continuous usage at the required flow rate (10 l min^−1^), the maximum humidity drops to <90% in 1 h owing to a decrease in the efficacy of humidification. Our current configuration is a 6′ long 3′′ diameter pipe for the reservoir, a 55′′ column of water and a flexible hose terminating at the bottom of the water reservoir in an aquarium airstone (¾ × ¾ × 2′′) for the air path, which yields a maximum RH of ∼99% and a >2 h drop to 95%.

For crystal manipulation under the humid flow, initially the crystal cover slips were simply placed on the microscope stage at the output of the humidity controller and the vials were held in the humidity path with clay. Subsequently, an adaptor was designed with a platform for crystal cover slips and a coupling to the output port of the humidity controller. The design was created in *Google SketchUp* and three-dimensionally printed online, first using standard white polyamide plastic (http://www.sculpteo.com). The granularity of the polyamide plastic made it difficult to visualize the crystals unless raised cover slips were used (*e.g.* EasyXtal plates, Qiagen), so we later switched to transparent resin (http://www.i.materialize.com). With the adaptor in place, a humid flow rate of 10 l min^−1^ was sufficient to establish a humid envelope covering the crystal cover slips. The adaptor also includes a port for vial mounting, with a cavity to hold a small rare-earth magnet that facilitates positioning the vial in the port (1/4′′ diameter × 1/8′′ thickness; K&J Magnetics; http://www.kjmagnetics.com). With the exception of the ‘Vial Mounting Fine Tuning’ crystals (Supplementary Table S1^1^), the adaptor was used for each of the vial-mounted crystals described. For information on acquiring the adaptor, please contact the authors.

#### 2.4.2. Vial mounting   

**Figure 2:**
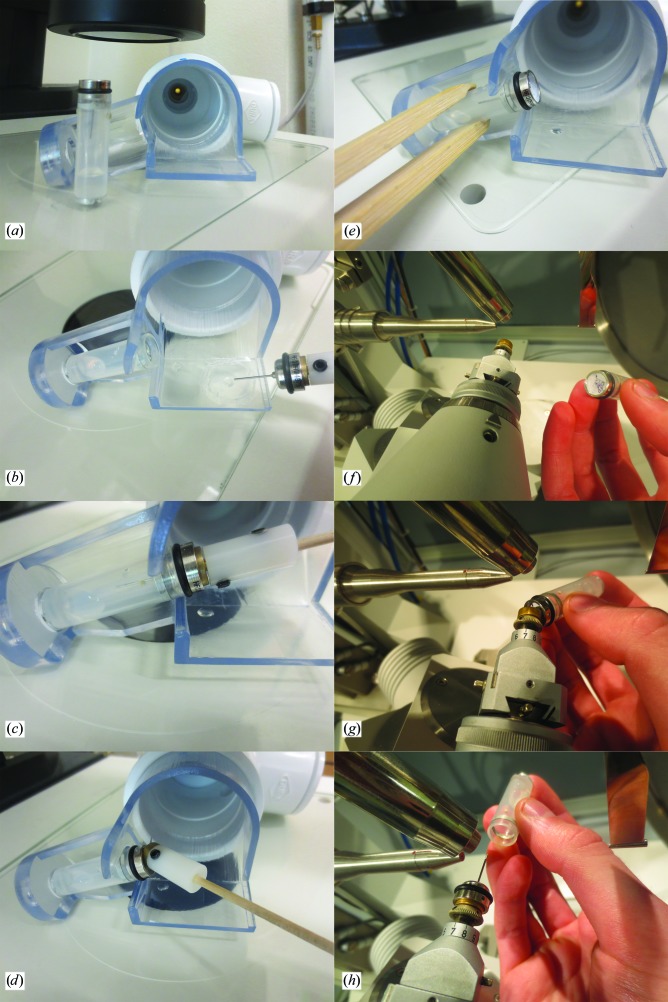
Workflow detail for vial mounting. (*a*) Humidity controller with adaptor in place sitting on the mounting microscope. A vial has been fitted with a crystal cap and O-ring and includes 500 µl cryosolution. (*b*) The vial and the crystal-growth coverslip are placed in the adaptor under humid flow. The crystal is transferred from the growth drop to the cryosolution and soaked for ∼2 min. (*c*) The crystal is looped and transferred to the vial. (*d*) The wand is removed from the vial. (*e*) The vial is removed from the adaptor with wooden tweezers and placed near the diffractometer in advance of cooling. After an equilibration period (2 min to overnight) the crystal is mounted directly on the cryostream. (*f*) The goniometer is positioned so the crystal will be mounted horizontally and the vial is brought towards the goniometer by hand (shown) or with wooden tweezers. (*g*) Mounting starts with the crystal cap and the vial is rotated into place. Although only one hand is shown (for clarity), this is usually performed with two hands for stability. (*h*) The vial is removed along the axial axis. A finger from the other hand can be placed on the crystal cap, ensuring that the loop stays in place. We try to limit the total time of the vial in the cold stream (*e* and *f*) to <2 s. The exact details of the mounting procedure will depend on the strength of the magnet on the goniometer head.

For vial mounting, standard CryoVials (Hampton Research; MiTeGen, Ithaca, New York, USA) were prepared by plugging the liquid-nitrogen escape holes with a small amount of clay or adhesive to prevent diffusion of humid air out of the vial and very lightly greasing the rim of the vial opening. Crystal caps were prepared by placing an O-ring on the cap to rest between the cap and the vial rim. Cryosolution (500 µl) was pipetted into the vial and the humid flow was set to the humidity value of the cryosolution predicted by Raoult’s law (Wheeler *et al.*, 2012 ▶). The vial-mounting procedure then proceeded as shown in Fig. 2 ▶.

To maintain a uniform and constant humidity in the vial, the seal between the crystal cap and the vial is important. Ideally, the humidity at the position of the crystal should be identical to the humidity at the surface of the liquid in the vial. For an open vial with 500 µl of water at 25% RH (294 K), we measured the evaporation rate (gravimetrically) to be 3 mg h^−1^ or 3 µl h^−1^. Assuming one-dimensional diffusion (along the vial cylindrical symmetry axis) at steady state with a linear water concentration between the surface of the water (100% RH or 23.0 g m^−3^; http://www.tis-gdv.de/tis_e/misc/klima.htm) and the mouth of the vial (25% RH or 5.7 g m^−3^) also yields a rate of 3 µl h^−1^ at 298 K and 1 atm. It is known that very small changes in humidity can produce measurable changes in unit-cell parameters. For example, oxidase crystals show a dependence of the *c* cell edge of up to 2.5 Å per 1% RH (Kiefersauer *et al.*, 2000 ▶). With our cryopins and 500 µl of liquid in the vial, the crystal sits 9 mm from the surface of the liquid or about one third of the way to the mouth of the vial. Assuming a surface vapor pressure equivalent to that of pure water and a target humidity drop of <1%, we need the water vapor gradient, and therefore the evaporation rate, to be reduced by a factor of at least 75/3, or 25. With the pin only, the evaporation rate is reduced by only a factor of four relative to the open vial. We therefore investigated methods of sealing the cap–vial junction. Two approaches were used. Initially, the junction was sealed after inserting the cap by wrapping crystal-mounting clay (Hampton Research) around a thin section of the vial, covering the cap–vial seam, which reduced the evaporation rate by a factor of ∼700. However, this clay-sealing approach requires substantial contact between fingers, the vial and the cap, which appears to upset the thermal equilibrium of the vial–cap system and hence the vapor equilibrium of the crystal and air inside, increasing the unit-cell variation. Therefore, a method requiring less handling was sought. We tested washers, gaskets and O-rings of many different materials and sizes with and without vacuum grease before adopting a 3/8′′ internal diameter × 1/16′′ thick O-ring (http://amazon.com; nitrile rubber, 50 A durometer hardness), which was placed over the cap. A small amount of vacuum grease was used to seal the interface between the O-ring and the vial. The O-ring with grease reduces the evaporation rate of water by a factor of ∼170, ensuring that the humidity drop between the solution and the crystal is ∼0.2% RH. Improvement of the sealing mechanism between the vial and the cap is ongoing. Additionally, we fashioned a wooden-handled cryowand using a magnetic base (Hampton Research) and modified some bamboo toast tongs (Harold Import Co., Lakewood, New Jersey. USA) by sanding a groove to accept the CryoVial. With the O-ring sealing mechanism and the wooden-handled tools, the complete vial-mounting process can be completed without direct contact between the experimenter’s fingers and either the cap or the vial.

### 2.5. Conditions for specific experiments   

#### 2.5.1. Air-path dependence   

Crystals were soaked in the respective cryosolutions for 2–3 min. Five different mounting microscope–cooling medium arrangements were used to achieve different air-path lengths: 0 m (using plunge-cooling with the direct-to-oil technique), 0.25 m (microscope to liquid-nitrogen dewar), 1.2 and 3 m (microscope to cryostream) and 5 m (3 m microscope position with extra distance in walking to the diffractometer). Crystal characteristics and ambient humidities were as follows. Thaumatin: bipyramids with base of 140–200 µm, RH = 24–31%. Lysozyme: blocks with 100–350 µm edges, RH = 26–30%. Thermolysin: rods with diameters 100–200 µm, RH = 23–29%. For all crystals, the ambient temperature was 294–297 K.

#### 2.5.2. Ambient humidity dependence   

Thaumatin crystals were soaked in the cryosolution [35%(*w*/*v*) EG, 0.8 *M* potassium sodium tartrate] for ∼2 min. The 0.1 mm loops were Hampton Research ‘0.1–0.2 mm’ loops, measured with an inside minor axis of 110–118 µm. The 0.05 mm loops were ‘0.05–0.1 mm’ loops measured with an inside minor axis of 70–75 µm. The ambient temperature was 294–297 K. A small room humidifier was used for the highest humidity data points. The crystals were 130–190 µm in size and the average crystal size for each data point was 162–173 µm.

#### 2.5.3. Crystal size dependence   

Thaumatin crystals were soaked in cryosolutions for ∼2 min. The vial-mounted crystals were O-ring sealed and equilibrated at room temperature (294 K) overnight (∼15 h). Two ranges of loop sizes were used to roughly maintain the relative volume of extra solution. For crystals larger than 120 µm ‘0.1–0.2 mm’ loops were used (125–155 µm minor-axis inner diameter). For crystals 120 µm and smaller ‘0.05–0.1 mm’ loops were used (70–80 µm minor-axis inner diameter). The ambient humidities for each series are as follows: vial mounting, 22–24% RH; 1.2 m microscope distance, 20–23% RH; 3.0 m microscope distance, 18–20% RH; plunge-cooling, 24–27% RH.

#### 2.5.4. Cryostream flow rate   

Thaumatin crystals were soaked in the cryosolution [35%(*w*/*v*) EG, 0.8 *M* potassium sodium tartrate] for ∼2 min, vial-mounted, clay-sealed and equilibrated at room temperature (294 K) overnight (∼15 h). The cryostream velocity was controlled by varying the nitrogen flow rate, with both the sample and the shield set to the same value. The set temperature was 120 K, since the more typical 100 K could not be achieved with the lower flow rates. The crystal sizes were 140–210 µm.

#### 2.5.5. Cryosolution soak time   

Thaumatin crystals were soaked in the cryosolution [35%(*w*/*v*) EG, 0.8 *M* potassium sodium tartrate] for beween 30 s and 5 min and were plunge-cooled in liquid nitrogen as described above. The crystal sizes were 150–190 µm.

#### 2.5.6. *F*_o_ − *F*_o_ map   

Thaumatin crystals were soaked in cryosolution [35%(*w*/*v*) EG, 0.8 *M* potassium sodium tartrate] with and without 0.3 *M* KCl or NaCl for 8 min, mounted in a vial and equilibrated for 2 min. Crystals were then either mounted directly in the cryostream from the vial or removed from the vial at the microscope (3 m microscope distance) and then directly mounted in the cryostream. X-ray data were collected with *CrysAlis^Pro^* (Agilent, Yarnton, England) and reduced with *SCALA* (Evans, 2006 ▶). Maps were calculated with the *CCP*4 package (Winn *et al.*, 2011 ▶) and the structures were refined (using PDB entry 1thw as the starting model; Ko *et al.*, 1994 ▶) using *REFMAC* (Murshudov *et al.*, 2011 ▶) and *Coot* (Emsley *et al.*, 2010 ▶). *F*_o_ − *F*_o_ maps were created as *F*_o_ (cryo + KCl or cryo + NaCl) − *F*_o_ (cryo only) in the *CCP*4 package. Data from the two crystals, with phases calculated from the refined coordinates for the cryo-only crystals (either vial-mounted or vial/direct-mounted), were combined in *CAD* and scaled with *SCALEIT*, and the map was calculated with *FFT*.

#### 2.5.7. Room-temperature controls   

Thaumatin crystals were loop-mounted directly from the growth drop, inserted into microRT tubes (Kalinin *et al.*, 2005 ▶; MiTeGen) containing a reservoir of the well solution and incubated for a few minutes at room temperature prior to collecting diffraction data.

### 2.6. X-ray data collection and analysis   

X-ray data were collected using an Agilent Xcalibur X-ray diffractometer with a Nova X-ray source and an Onyx detector (Agilent Technologies, Santa Clara, California, USA) using the following parameters: 50 kV, 0.8 mA, crystal-to-detector distance of 65.000 mm, θ (the detector angle) of 3.5°, oscillation width of 0.25°, number of frames = 2 × 6 separated by 90° and exposure time 15 s. Data were processed with *CrysAlis^Pro^* in Pre-experiment mode, which outputs unit-cell parameters, an estimate of the diffraction limit and mosaicity. In *CrysAlis^Pro^*, the ‘mosaicity’ is given as three components, *e*_1_, *e*_2_ and *e*_3_, which are the mosaicities in three directions defined in a coordinate system local to each reflection. *e*_1_ and *e*_2_ are the mosaicities (*i.e.* the angle subtended by the diffraction spots) in two orthogonal directions tangential to the Ewald sphere (on the image, *e*_2_ is the mosaicity along the direction radial from the beam center), while *e*_3_ is the mosaicity in a direction perpendicular to *e*_1_ and *S* − *S*_0_, which is roughly the mosaicity in the scanning direction. Here, *S* and *S*_0_ are the scattered and incident X-ray vectors, respectively (Kabsch, 2001 ▶). To allow the detection of asymmetric cooling effects, we started with unit-cell parameters unconstrained by space-group symmetry.

## 3. Results   

### 3.1. Dependence of unit-cell parameters on aspects of standard cryomounting   

#### 3.1.1. Transfer distance   

**Figure 3:**
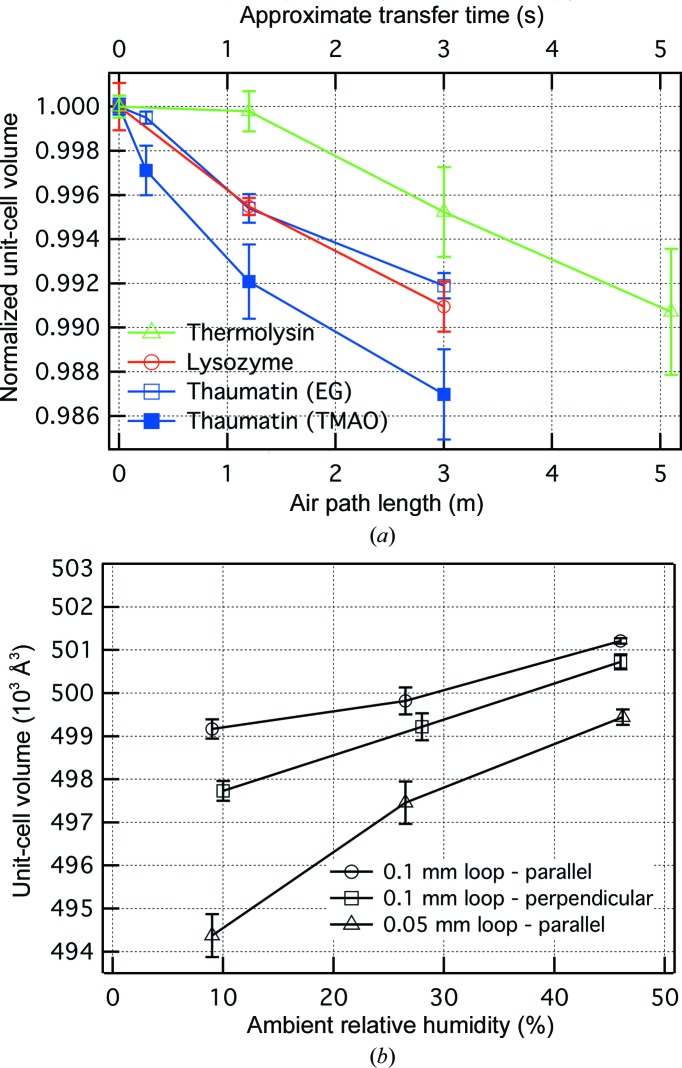
Unit-cell volume dependence on aspects of cryomounting. (*a*) Dependence on the air-path length between the mounting microscope and the cryogen for three different protein crystals. Each data set is normalized to the unit-cell volume for the direct-to-oil plunge technique (0 m). Each plotted point is an average of 4–9 crystals with error bars ± standard error of the measured volumes. The transfer time is only approximate: more accurate transfer times are given in Supplementary Table S1. (*b*) Dependence on the value of the ambient humidity and crystal orientation in the loop for thaumatin crystals. Crystals were direct-mounted from the 1.2 m microscope position. Each data point is an average of 4–5 crystals, and the error bars show the standard error. ‘Parallel’ and ‘perpendicular’ refer to the orientation of the long axis of the crystal relative to the long axis of the loop.

Fig. 3 ▶ shows the dependence of the low-temperature unit-cell volume on some parameters involved in standard direct mounting. Crystals of thaumatin, lysozyme and thermolysin all show smaller unit-cell volumes with longer transfer distances, which is consistent with a dehydration model in which the crystals dry out slightly during transfer to the cryogen (Fig. 3 ▶ *a*). The unit-cell volume dependencies are roughly −0.25% m^−1^ or −0.25% s^−1^, which for thaumatin corresponds to about −10^3^ Å^3^ s^−1^ (Fig. 3 ▶ *a*). Assuming a water-molecule volume of 30 Å^3^, 10^3^ Å^3^ corresponds to about 33 water molecules per unit cell. For crystals of the size represented in Fig. 3 ▶(*a*) (140–200 µm) this corresponds to a flux of water molecules out of the surface of the crystal in the range of ∼30–40 water molecules per Å^2^ per second. The measured rate of diffusion from a macroscopic flat surface of cryoprotective solution is about three times lower, or ∼10 molecules per Å^2^ per second, while a simple diffusion model for the evaporation of water from a stationary spherical drop of cryosolution of similar volume to these crystals predicts a flux of ten times higher, or ∼10^2^–10^3^ water molecules per Å^2^ per second. We expect the flux from the loop-mounted crystal to be lower than the flux from a pure solution drop, since (i) an exposed crystal may only lose water from the solvent channels, which occupy only some fraction of the surface, and (ii) any extra solution present during crystal mounting will act as a buffer. Therefore, the factor-of-ten rate reduction from the crystal relative to a pure solution drop is reasonable.

#### 3.1.2. Ambient humidity, loop size and orientation of crystal in the loop   

For thaumatin, we also investigated the dependence on ambient humidity, as shown in Fig. 3 ▶(*b*). The direct-mounted unit-cell volume decreases with lower ambient humidity, which is also consistent with a dehydration model. The relative humidity of the thaumatin EG-based cryosolution is about 80%. Since the evaporation rate is proportional to the humidity difference between the solution and the environment, lower humidity should cause the evaporation of more water during the same time period. Over a nine-month period the relative humidity in our laboratory varied from 9 to 50%, which judging from Fig. 3 ▶(*b*) could produce a 1% range in thaumatin unit-cell volumes.

Three different loop sizes/crystal orientations were used. For the parallel crystal orientation, the smaller loop yielded smaller unit-cell volumes at all humidities, with volume differences of 1000–5000 Å^3^. The smaller loop usually brings along less extra solution when looping, increasing the sensitivity of the crystals to dehydration. The 0.1 mm/perpendicular orientation had intermediate unit-cell volumes. While crystals in this orientation appeared to have intermediate amounts of extra solution, they will also sit differently in the cryostream during cooling. Both of these aspects, dehydration and orientation relative to the cryostream, could contribute to the intermediate volumes of crystals in the perpendicular orientation.

#### 3.1.3. Crystal size   

**Figure 4:**
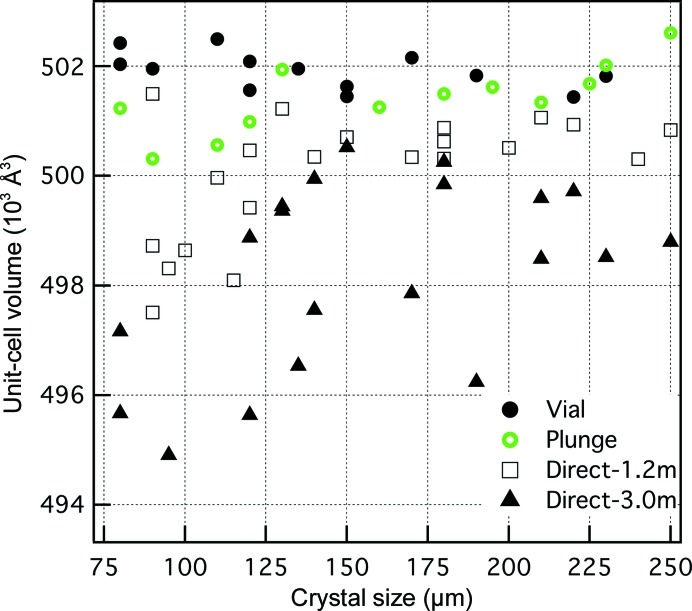
Dependence of the low-temperature thaumatin unit-cell volume on crystal size for four different cryomounting arrangements. All crystals were soaked in cryosolution (EG) for ∼2 min. Vial: crystals were equilibrated overnight in the vial and then mounted into the cryostream from the vial. Plunge: crystals were plunge-cooled in liquid nitrogen. Direct: crystals were directly mounted into the cryostream from two different microscope–cryostream distances.

Fig. 4 ▶ shows the dependence of unit-cell volume on thaumatin crystal size. For standard direct mounting, smaller crystals yield smaller unit-cell volumes. This general behavior was true for both cryoprotective agents tested, EG (Fig. 4 ▶) and TMAO (Supplementary Table S1), and can be explained by a dehydration model since the volume of water exiting the unit cell should depend directly on the surface area:volume ratio of the crystal, which is larger for smaller crystals. The size dependence is most clear for the microscope positioned at 1.2 m, immediately adjacent to the diffractometer. For the larger microscope distance (3 m; sitting in on a table in the diffractometer room) the size dependence is still evident, but there is greater scatter in the unit-cell volumes. For plunge-cooling the size dependence is much smaller over this crystal size range, as would be expected in a dehydration-based model owing to the fivefold to tenfold shorter exposure time than in direct mounting.

#### 3.1.4. Cryostream velocity and cryosolution soak time   

Although the size dependence for thaumatin crystals in Fig. 4 ▶ can be understood qualitatively with a dehydration model, other factors should be considered, including the cooling rate. For gas-stream cooling, cooling times are proportional to *L* ^3/2^, where *L* is a characteristic length of the crystal (Kriminski *et al.*, 2003 ▶). Thus, over the factor of three range of crystal sizes here, the smallest crystals should cool about five times more rapidly than the largest crystals. However, it is unclear whether higher cooling rates should yield larger or smaller unit-cell volumes. For very slow cooling ice may form and increase the unit-cell volume, but there is no evidence from the diffraction patterns of ice formation in the largest crystals. With complete vitrification, higher cooling rates could limit relaxations associated with thermal contraction, increasing the unit-cell volumes, which is the opposite of what we observe.

**Figure 5:**
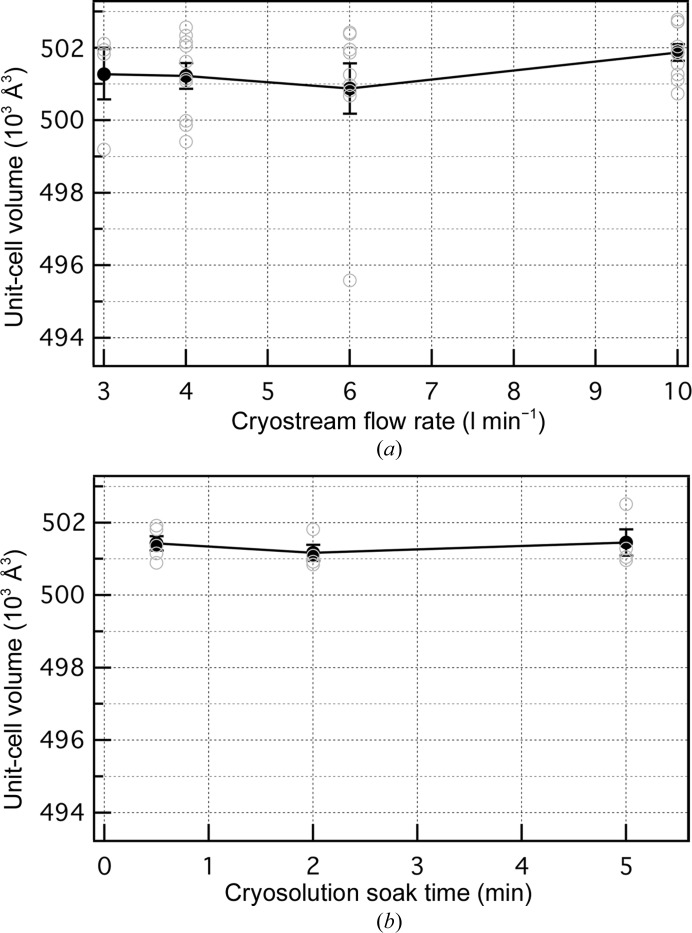
(*a*) Dependence of the thaumatin unit-cell volume on the flow rate of the cryostream. Both the sample and shield flow were set to the value on the horizontal axis. Each data point (black) is an average of 4–10 crystals (open gray circles); error bars show ± standard error. (*b*) Dependence of the thaumatin unit-cell volume on cryosolution soak time. Each data point (black) is an average of 4–5 crystals (gray); error bars show ± standard error. The figure shows that there is not a systematic dependence of the unit-cell volume on either the cryostream flow rate or the cryosolution soak time.

The cooling rate is also proportional to *u* ^−1/2^, where *u* is the relative velocity between the gas stream (Kriminski *et al.*, 2003 ▶). We therefore altered *u* by changing the flow rate of the nitrogen-gas stream by a factor of three and used vial-mounted crystals as a separate test of the effect of the cooling rate (Fig. 5 ▶ *a*). However, if the size dependence in Fig. 4 ▶ is a cooling-rate effect one would expect a negative slope in Fig. 5 ▶(*a*), and this is not the case.

The rate of diffusion of cryoprotective agent into the crystal should also be considered. Although our standard cryosolution soak time (2 min) is relatively short, we tested different soak times (30 s and 5 min) and found no effect on the unit-cell volumes (Fig. 5 ▶ *b*). Hence, the ∼0.6% larger unit-cell volume for the 250 µm crystals than for the 80 µm crystals does not appear to be owing to incomplete diffusion of cryosolution into the larger crystals.

Therefore, for thaumatin crystals neither cooling rate nor cryosolution soak time appear to affect the unit-cell volume, at least over the ranges tested, and the crystal size dependence of Fig. 4 ▶ is likely to be a dehydration effect. It is important to remember that cooling rate and soak time may be relevant for other crystals.

### 3.2. Unit-cell variation for alternative cryomounting procedures   

Alternative methods of crystal mounting that may limit dehydration were investigated. We first tested two variations on traditional plunge-cooling.

#### 3.2.1. Variations on plunge-cooling: direct-to-oil transfer   

One approach to limit dehydration is to use a kinetic barrier, which we implemented *via* a direct-to-oil mounting method in which a drop of oil was placed in touch with the drop of aqueous cryosolution containing the crystal. Using a loop, the crystal was pushed directly into the oil from the cryosolution, never coming directly in contact with the ambient air. The crystal was then looped (without removing any of the aqueous cryosolution remaining around the crystal) and plunged into liquid nitrogen, with the oil reducing the rate of water loss from the crystal during the brief ∼0.5 s transfer to the dewar of nitrogen (Juers & Matthews, 2004 ▶). This approach yielded high repeatability (σ_vol_ = 248 Å^3^ for a sample size of seven thaumatin crystals of 160–190 µm using the EG-based cryosolution; Supplementary Table S1) and some of the larger unit-cell volumes that we measured (501 665 Å^3^), consistent with the notion that little water is lost during mounting. (This can be compared with a similar sample for standard plunge-cooling: σ_vol_ = 363 Å^3^ for a sample size of seven thaumatin crystals of 140–190 µm; Supplementary Table S1.)

#### 3.2.2. Variations on plunge-cooling: plunge-cooling at high humidity   

Another approach is to adjust the humidity of the ambient air. We tested this method by equilibrating crystals with the cryosolution (5 min in 3.5 *M* TMAO) and then moving them into a cold room prior to plunging them into liquid nitrogen. Our cold room does not have humidity control, but typically has high relative humidity (90–95%). Cold-room mounting also yielded high repeatability (σ_vol_ = 295 Å^3^ for a sample size of eight thaumatin crystals of 150–170 µm; Supplementary Table S1). An additional benefit of mounting in the cold room is reduction of the vapor pressure of water, which reduces the kinetics of evaporation of water from the crystal.

#### 3.2.3. Vial mounting with humid flow   

To improve the reproducibility of direct mounting, vial mounting with humid flow was developed, which has three basic components: crystal manipulations under an adjustable humid flow, transfer of the crystal to a vial containing the crystal soaking solution and direct transfer of the crystal from the vial to the cryostream. In fine-tuning some aspects of the technique, we mounted nine sets of 5–9 thaumatin crystals, with each set having slightly different experimental conditions (Supplementary Table S1). The unit-cell variation of these sets ranged from σ_vol_ = 114 to 757 Å^3^, with an average (〈σ_vol_〉) of 324 Å^3^ or 0.06% of the unit-cell volume (about two times smaller than the direct-mounted variation).

Once developed, the vial-mounting procedure was then used for the larger size range of thaumatin crystals (80–230 µm) shown in Fig. 4 ▶ and Table 1 ▶. The vial-mounted crystals have lower unit-cell variation than either the direct-mounted or the plunge-cooled crystals and show the lowest mosaicities of the crystals tested.

For comparison, crystals of thermolysin, the system with the largest unit-cell volume variation in direct mounting (Supplementary Table S1), were also vial-mounted. With longer soaks (1 h), two sets of crystals yielded unit-cell variations of σ_vol_ = 680 and 890 Å^3^ (0.07 and 0.09%), which are 2–3 times smaller than the direct-mounted variation (Supplementary Table S1).

## 4. Discussion   

### 4.1. Dehydration during standard cryomounting reduces unit-cell volumes and can increase unit-cell variation   

The results in §[Sec sec3]3 indicate that during standard cryomounting there is a systematic dependence of the low-temperature unit-cell volume on dehydration, which has been well known since the beginning of macromolecular crystallo­graphy to impact crystal packing, crystal order (*i.e.* mosaicity and diffraction limit) and unit-cell parameters (Kiefersauer *et al.*, 2000 ▶; Russi *et al.*, 2011 ▶; Jaskolski *et al.*, 2014 ▶). In our experiments, the relative humidities of the cryosolutions are 80–90% RH, while the ambient humidity is 10–50% RH. Brief exposure of the crystal to this ambient air during cryomounting reduces low-temperature unit-cell volumes. Variation of experimental factors that impact exposure of the mounted crystal to ambient air will then lead to unit-cell variation, including transfer distance, ambient humidity, crystal size and loop size. Crystal orientation in the loop may also be important, as well as other parameters that are not directly investigated here. The amount of variation observed for the thaumatin crystals is ∼1% in unit-cell volume, corresponding to unit-cell parameter differences of ∼0.2–0.5 Å.

### 4.2. Unit-cell parameter differences allowable for effective isomorphism   

A unit-cell parameter shift of 0.5 Å may seem relatively small. We therefore consider here what level of unit-cell variation is allowable for multicrystal techniques, considering successively MIR, SAD and *F*_o_ − *F*_o_ electron-density maps. For MIR, differences in unit-cell dimensions of up to *d*_min_/4 are acceptable (*e.g.* 0.5 for 2.0 Å resolution data; Garman & Murray, 2003 ▶). For anomalous scattering, the tolerable difference can be smaller. In a recent investigation of multiple crystal methods for SAD, a clustering analysis was used to merge data from multiple cryocooled crystals (Giordano *et al.*, 2012 ▶). Four different crystal systems were tested, and in each case clusters of similar crystals were identified. Merging data from crystals within the same cluster improved the anomalous signal, while in most cases merging data from crystals in different clusters degraded the anomalous signal. Most of the clusters showed unit-cell parameter ranges of less than 0.5 Å (nine of the 11 clusters identified across the four different crystal systems). For the specific case of thaumatin 13 crystals were tested and two clusters of crystals were identified with unit-cell parameter ranges of 0.12 Å for *a* and *b* and 0.23 and 0.24 Å for *c* (Giordano *et al.*, 2012 ▶). Hence, for thaumatin crystals a unit-cell parameter difference of 0.5 Å would be likely to render the two crystals unmergable for the purpose of using anomalous scattering for phasing.

**Figure 6:**
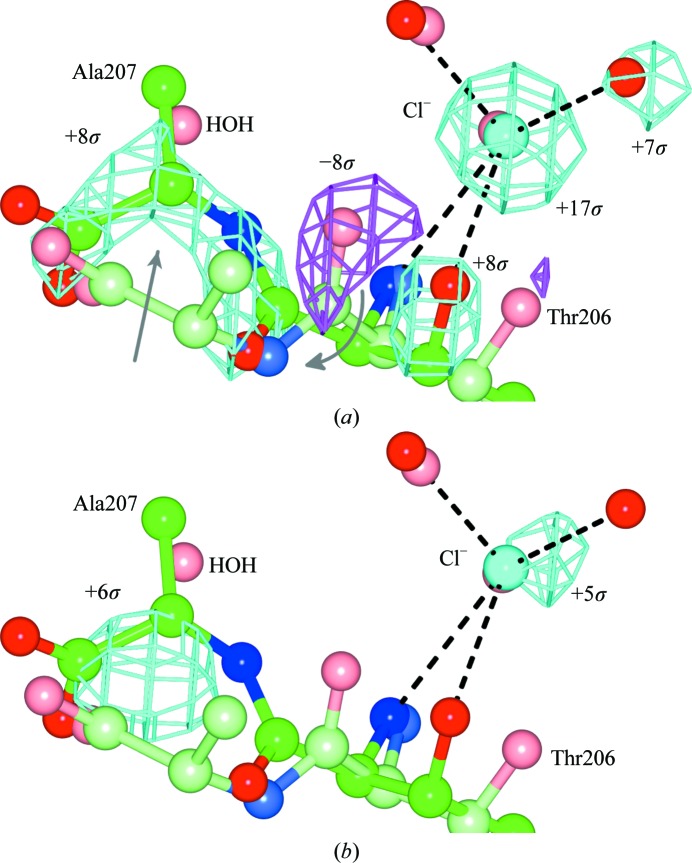
*F*_o_ − *F*_o_ maps for a soak of thaumatin crystals in 300 m*M* KCl illustrating tolerable unit-cell differences. The maps are contoured at ±4.5σ (cyan/magenta). Coordinates are refined coordinates (bright green/red/blue, cryo + KCl soak; light green/red/blue, cryo-only soaks). The maps were calculated as *F*_o_ (cryo + KCl soak) − *F*_o_ (cryo only). (*a*) Map between two vial-mounted crystals with unit-cell parameter differences of −0.06 Å (*a* and *b*) and 0.01 Å (*c*). Phases are from the refined coordinates for the vial-mounted cryo-only crystal. The map clearly indicates the location of a bound ion (modelled as a Cl^−^), as well as a small rearrangement of the two C-terminal residues (Thr206 and Ala207). (*b*) Map between the vial-mounted cryo + KCl crystal and a direct-mounted cryo-only crystal, with unit-cell parameter differences of 0.23 Å (*a* and *b*) and 0.49 Å (*c*). Phases are from the refined coordinates for the direct-mounted crystal. The peak for the bound ion is reduced from 17σ to 5σ and the map is much less informative. This figure was prepared with *CCP*4*mg* (McNicholas *et al.*, 2011 ▶).

*F*_o_ − *F*_o_ electron-density maps can be very useful for detecting changes in macromolecular structure such as small conformational changes and ligand binding. However, the effective use of *F*_o_ − *F*_o_ maps requires isomorphism between the two crystals. As an example, we soaked a thaumatin crystal in cryosolution with and without added 300 m*M* KCl to look for ion-binding sites. In one case, two crystals were vial-mounted, yielding unit-cell parameter differences of −0.06 Å in *a* and *b* and 0.01 Å in *c*. The *F*_o_ − *F*_o_ map was of great use, showing a 17σ peak (modelled as a Cl^−^ ion), some ∼8σ peaks indicating a small rearrangement of the C-terminus and some ∼7σ peaks indicating reduced occupancy of a secondary tartrate molecule. In another case a crystal with no added KCl was direct-mounted, yielding unit-cell parameter differences of 0.23 Å in *a* and *b* and 0.49 Å in *c*, and the *F*_o_ − *F*_o_ map was less useful as the chloride peak was reduced to 5σ (Fig. 6 ▶). Thus, the 0.2–0.5 Å shift in unit-cell parameters from dehydration reduced the intensity of the Cl^−^ peak from 12σ above the background shifting level to 1σ above the background shifting level. The same test with 300 m*M* NaCl gave a similar but less pronounced result, with the chloride peak appearing at 14σ *versus* 9σ in the vial–vial *versus* vial–direct comparisons (Table 2 ▶).

In summary, while a small unit-cell parameter shift (*i.e.* 0.5 Å) may be tolerable for some purposes, it will not be for others. Any procedure or technique that can improve isomorphism even at the 0.5 Å level is likely to benefit structure determination and interpretation.

### 4.3. Improving isomorphism between multiple cryocooled crystals   

#### 4.3.1. General guidelines for standard cryomounting   

Some simple guidelines may be considered to improve the repeatability of standard cryomounting, with the overall aim of ensuring that the volume of water leaving the unit cell is consistent from sample to sample. Transfer times, crystal sizes and the orientation of the crystal in the loop should be consistent. Loop size relative to the crystal should leave some external solution, and extensive wicking, blotting and other procedures should be avoided. [If repeatability is NOT a concern, the removal of external solution may be beneficial by increasing signal to noise and reducing the required cryoprotectant concentration (Kitago *et al.*, 2005 ▶)]. Care should be taken to limit the path length travelled by the crystal through the dry cryostream (*i.e.* move the crystal perpendicular to the velocity of the cryostream). Ideally, the physical environment of the room where mounting is carried out should be controlled at constant humidity. The above considerations should help any particular researcher achieve higher repeatability of unit-cell parameters for particular crystals of interest using standard cryomounting techniques.

Many of the aspects of the cryomounting process, however, are difficult to control and will affect the amount of dehydration. One may therefore expect that crystals of different sizes, with different cryosolutions (with different humidities), mounted on different days (with different ambient humidities), with different loops, in different orientations, by different people (who have different looping mechanics) will tend to have different unit-cell parameters and the differences may be too large to successfully merge data. If two laboratories are at different relative humidities, techniques that are effective in one laboratory may be ineffective in the other. Approaches that limit or control dehydration will be helpful for the development of a robust crystal-mounting procedure that is insensitive to variation in the above experimental conditions and achieves higher reproducibility of unit-cell parameters.

#### 4.3.2. Improved cryomounting techniques that limit dehydration   

*Plunge-cooling enhancements.* The simplest approach to limit water loss is to reduce the transfer time to cryogen, as in plunge-cooling, which reduces the dependence of unit-cell volume on crystal size (Fig. 4 ▶). However, plunge-cooled crystals still undergo a short (∼0.25 m and ∼0.5 s) exposure to ambient air and exhibit measureable unit-cell variation (Table 1 ▶). Enhancements to plunge-cooling explored here are direct-to-oil transfer and plunge-cooling at high humidity. These two approaches show high repeatability of unit-cell parameters in thaumatin crystals, and should be more resistant to dehydration effects than standard plunge-cooling (§[Sec sec3.2.1]3.2.1).

*Vial mounting with humid flow.* Both of the above approaches require dunking into a liquid cryogen, and involve media (oil) or temperatures (4°C) with which the crystal may not be compatible. We therefore sought a more general method of limiting water loss during cryomounting which would be applicable at room temperature without the use of oil. Vial-mounting with humid flow allows crystal transport to the cryostream, in principle over arbitrary distances and times, at the same time keeping the crystal in equilibrium with its growth or soaking solution, thereby limiting water loss during cryotransfer and reducing dehydration-based variation.

The vial-mounting method turns a strongly positive dependence of thaumatin unit-cell volume on crystal size into a weakly negative one and can reduce the unit-cell variation (Fig. 4 ▶). The vial-mounted thaumatin crystals show greater unit-cell parameter reproducibility for the large size range of crystals than either plunge-cooled or direct-mounted crystals (Table 1 ▶). Although isomorphism involves more than unit-cell parameters (molecular orientation in the unit cell is also important), it is of note that as a group the vial-mounted crystals have lower unit-cell parameter standard deviations than either cluster for which data were successfully merged by Giordano *et al.* (2012 ▶) for SAD phasing (Table 1 ▶).

Additionally, the vial-mounted crystals show lower mosaicities (by 10–15%) on average than either the direct-mounted or the plunge-cooled crystals, and the lowest discrepancy between the *a* and *b* unit-cell edges. The vial-mounted crystals thus appear to be more highly ordered. A possible explanation is that the initiation of diffusion processes by both direct-mounting and plunge-cooling upon exposure to ambient air may create concentration gradients within the crystal, leading to less uniform cooling.

The instrument unit-cell volume uncertainty as reported by *CrysAlis^Pro^* for the thaumatin crystals tested is usually about 50 Å^3^ or 0.01%, which is about seven times smaller than the typical variation for the vial-mounted crystals. It may therefore be possible to further improve the measurable reproducibility. The source of the baseline variation for vial-mounting could be inherent room-temperature variation that exists just prior to cooling, or variation in the cooling process itself; for example, the orientation of the crystal relative to the cryostream. Room-temperature unit-cell volumes measured with microRT tubes showed a variation of σ_vol_ = 689 Å^3^ (Supplementary Table S1), so we cannot exclude an inherent room-temperature variation immediately prior to cooling. Further experiments using vial-mounting or enhanced plunge-cooling may help to clarify these issues.

### 4.4. Parameters involved in vial-mounting   

While vial-mounting with humid flow can improve the reproducibility of cryocooling, it also introduces new variables to be considered when designing and carrying out cryocooling protocols.

#### 4.4.1. Humidity and flow rate of humid flow   

The characteristics of the humid flow can be adjusted. The humidity setting used here was based on Raoult’s law and was confirmed using a humidity probe over a centrifuge tube filled with cryosolution. The flow rate was chosen empirically to give a humid envelope that included the vial and the crystal drop. Other humidities and flow rates could in principle be used.

#### 4.4.2. Composition of the solution in the vial   

In most cases, we used a vial solution identical to the cryosoaking solution. However, this need not be the case, and the use of different soaking and vial solutions presents some new possibilities for cryocooling.

#### 4.4.3. Equilibration time of the crystal in the vial   

Initially, we chose a 2 min equilibration time since numerical solutions to the diffusion equation predict that a linear water-vapor gradient within the vial should dissipate in a few seconds. However, we found that changes in unit-cell volumes occurred on time scales longer than 2 min. Crystals larger than ∼150 µm showed ∼500 Å^3^ larger unit-cell volumes with overnight equilibrations, while the opposite effect occurred for smaller crystals. (Other experiments suggest that the ∼500 Å^3^ increase for large crystals occurs in <60 min.) Hence, the negative crystal size dependence for the vial mounts with overnight equilibrations shown in Fig. 4 ▶ is stronger for 2 min equilibrations. The source of the extended time dependence is not yet known, but it has been pointed out that humidity-dependent crystal-packing changes can happen slowly (Sanchez-Weatherby *et al.*, 2009 ▶). Practically, for many of our experiments we adopted a standard practice of setting up the vial mounts in the evening, equilibrating overnight and then collecting diffraction data in the morning. The longest equilibration that we tested was a few days, for which the crystal diffracted similarly to shorter equilibration. The maximum equilibration time without affecting the diffraction is probably similar to limiting times for crystals sitting in growth drops, depending mainly on the seal between the vial and the cap.

#### 4.4.4. Temperature of pin–vial assembly   

Because humidity is very sensitive to temperature (http://www.tis-gdv.de/tis_e/misc/klima.htm), the temperature of the pin–vial assembly can have a significant impact on the unit-cell volumes. We discovered this when trying to isolate a systematic difference in unit-cell volumes (up to 0.4%) achieved by two different experimenters. The unit-cell volume difference was traced to a 10°C difference in finger temperature. We hypothesize that warm fingers increase the temperature of the crystal cap, increasing the temperature of the gas in the vial, reducing the relative humidity in the vial, causing more water to evaporate from both the crystal and the solution in the vial and yielding smaller unit-cell volumes. Cold fingers have the opposite effect. We therefore used a wooden-handled wand and wood tweezers modified with a groove to accept a cryovial to limit heating or cooling of the crystal cap or the cryovial.

#### 4.4.5. Time of transfer in the cryostream   

Transfer of the vial into the cryostream cools the system, promoting condensation of water on the crystal, which increases unit-cell volumes and can cause ice. For the thaumatin crystals tested here, the thermal mass of the cryovial appears to give ample time (2–3 s) for mounting. We performed some tests (using a thermocouple located at the crystal position in a cryoloop) on vials dipped in liquid plastic or wrapped in rubber or fiberglass insulation, which increased the time available for mounting by twofold to fivefold. This could be necessary for crystals that are more sensitive to humidity than thaumatin crystals.

### 4.5. Added benefits of vial-mounting and humid flow   

Vial-mounting with humid flow represents a new approach to achieving cryoprotected crystals with the following additional technical benefits. Specific examples are given using thaumatin crystals.

#### 4.5.1. Crystal manipulations under humid flow   

Manipulating crystals in a controllable humid flow limits dehydration effects owing to environmental conditions while harvesting and soaking crystals. Since the rate of water evaporation is proportional to the humidity difference between the crystal solution and the environment, the use of humid flow can reduce the evaporation rate several-fold, allowing more time for crystal manipulations. It would be beneficial for a humidity controller with an easily positioned output port to be standard equipment for any crystallography laboratory. Similar considerations apply for the development of robotic mounting devices (Deller & Rupp, 2014 ▶). To maximize reproducibility, humidity control needs to be an integral part of structure-determination pipelines.

As an example, we tried removing solution external to the crystal, which is known to increase the signal to noise (Kitago *et al.*, 2005 ▶) and in some cases to be adequate cryoprotection by itself (Pellegrini *et al.*, 2011 ▶). Thaumatin crystals were vial-mounted after blotting ten times (about 5 s) on the cover slip in humid flow, yielding an average unit-cell volume *V* = 501 594 (400) Å^3^ (Supplementary Table S1), which is within 300 Å^3^ of the expected volume for a vial-mounted crystal. In contrast, blotting in ambient humidity (27% RH) prior to direct-mounting (1.2 m microscope distance) yielded *V* = 495 278 (925) Å^3^, which is expected from Fig. 3 ▶(*a*) (Supplementary Table S1).

#### 4.5.2. Cryoequilibration *via* vapor diffusion   

**Figure 7:**
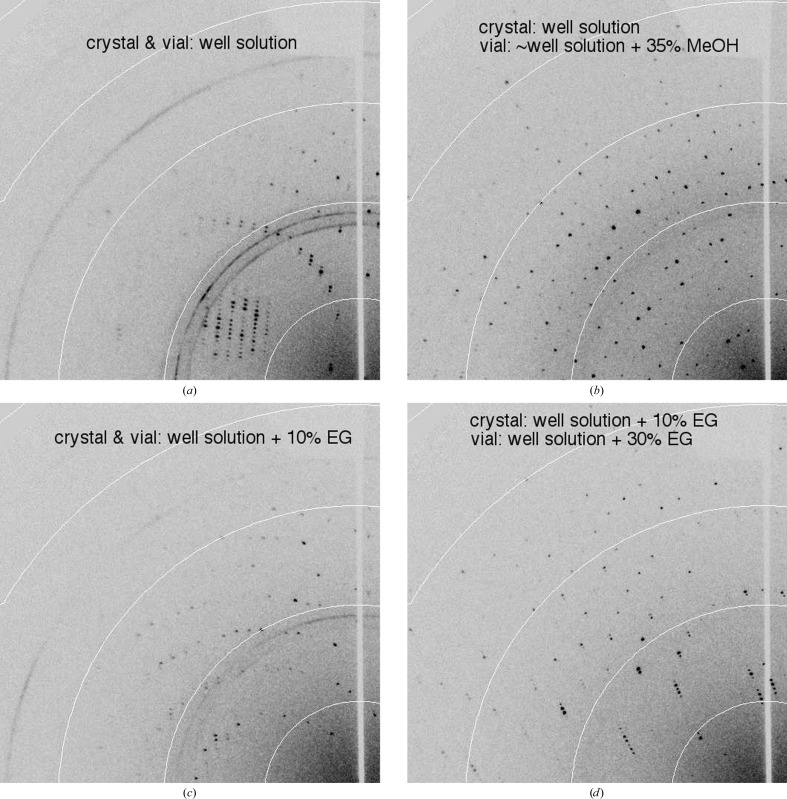
The use of vapor equilibration to achieve cryoprotection of thaumatin crystals. All crystals were mounted on the diffractometer directly from the vial as described in §[Sec sec2]2. (*a*, *b*) Crystals mounted directly from the growth drop and equilibrated in the vial over (*a*) the well solution for 45 min or (*b*) well solution (diluted to 80%) + 35%(*w*/*v*) methanol for 9 min. (*c*, *d*) Crystal soaked for 2 min in well solution + 10%(*w*/*v*) EG and equilibrated in a vial over (*c*) the same solution for 2 h or (*d*) well solution + 30%(*w*/*v*) EG for 45 min.

*Volatile cryoprotective agents.* Although difficult to work with, organic solvents have long been known to be useful for low-temperature crystallography (Petsko, 1975 ▶). With vial-mounting, the high vapor pressure of organic solvents can be turned into an advantage by equilibrating *via* vapor diffusion. Fig. 7 ▶ shows diffraction patterns of crystals that were looped directly from the growth buffer and then placed in a vial over the well solution either with or without methanol. Here, methanol is an adequate cryoprotective agent (Table 3 ▶) and no soaking was required for the equilibration. This approach has the advantage of requiring minimal handling and may be especially useful for fragile crystals and for those grown from conditions using organic solvents as precipitating agents.

*Nonvolatile cryoprotective agents.* Cryoequilibration *via* vapor diffusion can also be helpful for nonvolatile cryoprotective agents. In cases where crystals are grown from relatively low concentrations of precipitants that are natural cryoprotective agents (such as low-molecular-weight PEGs, sodium malonate and other salts), crystals could be looped directly from the growth drop into a vial containing a higher concentration of the precipitant. This will cause water transport out of the crystal and a gradual increase in cryoprotectant concentration in the crystal. An example is shown in Fig. 7 ▶ of a crystal soaked in 10% EG and then equilibrated against 30% EG to complete the cryoprotection. See also Table 3 ▶. This may be viewed as a complementary technique to simply directly soaking the crystal in the higher cryoprotectant concentration, since the vapor-equilibration case should be more dehydrating.

#### 4.5.3. Systematic cryoannealing   

There are many examples of the improvement of low-temperature diffraction *via* cryoannealing techniques (Yeh & Hol, 1998 ▶; Harp *et al.*, 1998 ▶; Hanson *et al.*, 2003 ▶; Juers & Matthews, 2004 ▶). Cryoannealing normally involves briefly blocking the cold stream or removing the crystal to some external solution for some period of time before remounting. Vial-mounting offers the possibility of demounting the crystal directly with a vial containing a solution of known humidity before remounting. We performed three tests of annealing with vial-mounting. In each case, five crystals were vial-mounted, equilibrated overnight, diffracted, demounted directly with the same vial, re-equilibrated for several hours and diffracted again. In each case, σ_vol_ decreased (1143 to 427 Å^3^, 468 to 355 Å^3^ and 545 to 395 Å^3^) and the average diffraction limit improved slightly (2.47 to 2.41 Å, 2.17 to 2.15 Å and 2.36 to 2.26 Å) (Supplementary Table S1).

## 5. Summary   

Reproducibility of cryocooling is an important aspect of macromolecular crystallography. The results presented here show that dehydration during crystal mounting can be a significant source of unit-cell variation. Implementation of a humid environment during crystal manipulation, combined with mounting procedures that reduce dehydration, can improve the reproducibility of cryomounting and offers some new approaches to crystal handling and cryoprotection in macromolecular crystallography.

## Supplementary Material

Data for all crystals analyzed.. DOI: 10.1107/S1399004714012310/tz5056sup1.xlsx

## Figures and Tables

**Table 1:** Statistics for cryomounted thaumatin crystals *a*, *b* and *c* are the edges for a unit cell unconstrained by the expected tetragonal symmetry of the thaumatin crystals. Vol, unit-cell volume. *e* refers to the average of the three components of the mosaicity output by *CrysAlis^Pro^* (*e*_1_, *e*_2_ and *e*_3_). Rows 1–4 show data for the crystal size series (Fig. 4 ▶). Rows 5–8 summarize the data reported by Giordano *et al.* (2012 ▶).

Data set	σ_vol_ (Å^3^)	σ_(*a*+*b*)/2_ (Å)	σ_*c*_ (Å)	〈Vol〉 (Å^3^)	〈(*a* + *b*)/2〉 (Å)	〈*c*〉 (Å)	Crystal sizes (µm)	*N*	〈*e*〉 (°)	〈|*a* − *b*|〉 (Å)
Vial	337	0.021	0.057	501910	57.81	150.14	80–230	13	0.48	0.020
Plunge	604	0.033	0.072	501427	57.84	149.91	80–260	13	0.55	0.029
Direct (1.2 m)	1241	0.050	0.135	499913	57.75	149.88	90–250	21	0.51	0.024
Direct (3.0 m)	1709	0.065	0.202	498247	57.69	149.73	80–250	20	0.56	0.042
Giordano *et al.*, cluster 1	783	0.041	0.069	504273	57.94	150.24	∼10–15	7	—	—
Giordano *et al.*, cluster 2	1284	0.055	0.109	502554	57.85	150.17	∼10–15	4	—	—
Giordano *et al.*, all data	1195	0.058	0.081	503705	57.91	150.22	∼10–15	13	—	—

**(a):** Data-collection statistics.

Mounting method	Vial	Vial	Vial	Vial/direct
Soak	Cryo + 0.3 *M* KCl	Cryo + 0.3 *M* NaCl	Cryo only	Cryo only
*a* (Å)	57.797	57.776	57.852	57.570
*b* (Å)	150.012	149.958	150.002	149.518
Multiplicity	6.1 (4.8)	5.2 (4.2)	6.0 (4.6)	5.6 (4.2)
Completeness (%)	99.3 (97.3)	98.5 (92.7)	99.3 (100.0)	99.8 (100.0)
〈*I*/σ(*I*)〉	37.1 (17.0)	31.5 (13.6)	37.5 (16.4)	30.1 (14.1)
*R*_meas_ (%)	3.6 (9.2)	4.0 (10.3)	3.6 (8.7)	4.7 (9.1)

**(b):** *F*_o_ − *F*_o_ map features.

	Vial (KCl) − vial	Vial (KCl) − vial/direct	Vial (NaCl) − vial	Vial (NaCl) − vial/direct
Δ*a*	−0.055	0.227	−0.076	−0.206
Δ*c*	0.010	0.494	−0.054	0.440
Map height at Cl^−^ (σ)	17	5	14	9
Map height at C-terminus	±8	6	±7	±4
Map height at tartrate	−7	−3	−6	−5
Map height at S—S shifting peaks	−4	−5	±6	±6

**Table 3:** Vial soaking/equilibration conditions and diffraction characteristics for in-vial vapor-diffusion equilibrations of cryoprotective agents

Soak	Vial equilibration	Unit-cell volume (Å^3^)	Diffraction limit (Å)	Mosaicity (°)
—	Well	510269	3.1 (+ ice)	0.67
—	Well (80%) + 35% MeOH	500895	2.0	0.53
Well + 10% EG	Well + 10%(*w*/*v*) EG	511929	3.2 (+ ice)	0.65
Well + 10% EG	Well + 30%(*w*/*v*) EG	500573	2.1	0.53

## Footnotes

1 Supporting information has been deposited in the IUCr electronic archive (Reference: TZ5056).
